# Supplementary material for: The Impact of Vegan Diet in the Prevention and Treatment of Type 2 Diabetes: A Systematic Review
Source: Nutrients. 2021 Jun 21;13(6):2123. doi: 10.3390/nu13062123 (PMC8235036; doi:10.3390/nu13062123)
Supplement: Supplementary file 1 [file nutrients-13-02123-s001.zip › nutrients-1240057-supplementary.pdf]

Supplementary Table S1. Changes in Fasting Plasma Glucose levels (mg/dL) by group assignment.

Reference	Intervention Group			Control Group			Between–Group Difference (Intervention–Control) Mean (95% CI)	*p*–Value
	Baseline	Final	Change	Baseline	Final	Change		
Nicholson (1999) [1]	193.32 (51.3)	139.50 (37.26)	−53.82	177.48 (29.34)	155.52 (3.6)	−21.96	−31.86*	<0.05
Barnard (2009 [2]	163.5 (7.6)	144.0 (7.7)	−19.5 (7.1)**	160.4 (5.8)	146.4 (8.03)	−14.0 (8.2)	−5.5 (−27.1 to 16.0)	0.61
Bunner (2015) [3]	160.6 (73.5)	134.6 (51.6)	−25.9 (65.0)	157.4 (60.5)	138.2 (51.3)	−19.2 (55.6)	−6.8 (−49.0 to 35.5)	0.75
Lee (2016) [4]	138.4 (52.4)	125.2 (38.0)	−13.2 (47.4)	126.3 (37.7)	126.3 (33.0)	0.0 (39.1)	−13.2	0.146
Barnard (2018) [5] median	125.0	109.0	−16.0	126.0	101.0	−12.5	7.5 (−14 to 44)	0.71
Wheeler (2002) [6] 10.5 h AUC (area under the curve) (mg%-h)	2442 (229)	2 075 (135)	−367	2179 (156)	2061 (166)	−118	−249	0.12

Data are presented as mean (SD, if available) unless otherwise indicated, **p* < 0.05, ***p* < 0.01, CI = Confidence Interval.

Supplementary Table S2. Changes in BMI (kg/m²) by group assignment.

Reference	Intervention Group			Control Group			Between-Group Difference (Intervention-Control) Mean (95% CI)	*p*-Value
	Baseline	Final	Change	Baseline	Final	Change		
Barnard (2009) [2]	33.9 (1.1)	32.3 (1.2)	−1.6 (0.3)**	35.9 (1.0)	34.8 (1.1)	−1.1 (0.3)**	−0.5 (−1.4 to 0.4)	0.25
Mishra (2013) [7]	33.5 (0.6)	32.0 (0.6)	−1.5 (0.2)**	34.0 (0.6)	34.0 (0.6)	0.0 (0.1)	−1.6 (−1.1 to −1.9)**	<0.001
Bunner (2015) [3]	35.9 (6.0)	33.5 (5.7)	−2.4 (1.5)**	36.2 (7.1)	36.0 (7.1)	−0.2 (1.2)	−2.2 (−3.2 to −1.2)**	<0.001
Lee (2016) [4]	23.9 (3.4)	23.5 (3.4)	−0.4 (0.9)**	23.1 (2.4)	23.0 (2.4)	−0.1 (0.6)	−0.3	0.092
Barnard (2018) [5]	34.9 (1.5)	32.6 (1.3)	−2.3 (0.4)**	33.0 (1.3)	31.5 (1.2)	−1.5 (0.2)**	−0.8 (−1.7 to 0.1)	0.075

Data are presented as mean (SD, if available) unless otherwise indicated, **p* < 0.05, ***p* < 0.01, CI = Confidence Interval.

Supplementary Table S3. Changes in Waist Circumference (cm) by group assignment.

Reference	Intervention Group			Control Group			Between-Group Difference (Intervention-Control) Mean (95% CI)	*p*-Value
	Baseline	Final	Change	Baseline	Final	Change		
Barnard (2009) [2]	110.8 (2.6)	106.6 (2.8)	−4.2 (1.0)**	112.3 (2.1)	110.5 (2.1)	−1.8 (0.8)*	−2.4 (−4.9 to 0.1)	0.06
Ferdowsian (2010) [8]	110.3 (1.9)	105.5 (1.9)	−4.7 (0.6)**	110.2 (2.7)	111.0 (2.8)	0.8 (0.6)	−5.5 (−7.3 to −3.7)**	<0.0001

Lee (2016) [4]	85.0 (9.8)	81.9 (9.9)	−3.1 (4.9)**	82.3 (7.5)	81.5 (7.9)	−0.8 (4.6)	−2.3	0.027

Data are presented as mean (SD, if available) unless otherwise indicated, *p < 0.05, **p < 0.01, CI = Confidence Interval.

Supplementary Table S4. Changes in Systolic Blood Pressure levels (mmHg) by group assignment.

Reference	Intervention Group			Control Group			Between-Group Difference (Intervention-Control) Mean (95% CI)	p-Value
	Baseline	Final	Change	Baseline	Final	Change		
Nicholson (1999) [1]	136.6 (10.0)	126.2 (14.9)	−10.4	149.5 (21.6)	130.6 (11.9)	−18.9	8.5	
Wheeler (2002) [6]	145 (4)	141 (3)	−4	145 (3)	140 (4)	−5	1	0.90
Barnard (2009) [2]	123.8 (2.5)	123.8 (2.4)	0.0 (2.0)	122.9 (2.1)	126.6 (2.4)	3.7 (1.9)	−3.7 (−9.2 to 1.8)	0.19
Ferdowsian (2010) [8]	118.8 (1.7)	118.8 (1.7)	0.0 (1.4)	116.4 (1.6)	122.1 (2.5)	5.7 (2.2)**	−5.7 (−10.6 to −0.9)*	0.03
Mishra (2013) [7]	127 (1.6)	124 (1.4)	−2.6 (1.0)*	127 (1.4)	123 (1.0)	−3.6 (1.1)**	0.9 (−2.1 to 4.1)	0.60
Bunner (2015) [3]	139.3 (17.0)	127.9 (22.6)	−11.5 (22.5)	144.4 (21.6)	140.1 (19.3)	−4.3 (15.0)	−7.2 (−20.6 to 6.2)	0.28
Lee (2016) [4]	125.1 (16.1)	126.1 (14.4)	1.0 (14.9)	128.1 (19.9)	126.6 (16.1)	−1.5 (18.7)	2.5	0.186
Barnard (2018) [5]	133.4 (2.3)	131.8 (4.1)	−1.6 (2.9)	126.8 (4.8)	119.7 (4.0)	−7.1 (3.9)	5.5 (−5.6 to 16.5)	0.31

Data are presented as mean (SD, if available) unless otherwise indicated, *p < 0.05, **p < 0.01, CI = Confidence Interval.

Supplementary Table S5. Changes in Diastolic Blood Pressure levels (mmHg) by group assignment.

Reference	Intervention Group			Control Group			Between-Group Difference (Intervention-Control) Mean (95% CI)	p-Value
	Baseline	Final	Change	Baseline	Final	Change		
Nicholson (1999) [1]	84.0 (4.5)	78.2 (9.7)	−5.8	86.0 (15.8)	75.4 (11.6)	−10.6	4.8	
Wheeler (2002) [6]	83 (2)	80 (2)	−3*	82 (2)	78 (2)	−4*	1	0.75
Barnard (2009) [2]	77.9 (1.6)	74.0 (1.4)	−3.9 (1.3)**	80.0 (1.5)	77.3 (1.5)	−2.7 (1.1)*	−1.2 (−4.6 to 2.2)	0.48
Ferdowsian (2010) [8]	80.5 (1.2)	80.1 (1.1)	−0.4 (1.1)	78.4 (1.3)	83.5 (1.5)	5.1 (1.2)**	−5.6 (−9.0 to −2.2)**	0.002
Mishra (2013) [7]	83 (1.1)	79 (1.0)	−3.5 (0.8)**	81 (0.9)	79 (0.8)	−2.6 (0.7)**	0.9 (−2.1 to 4.1)	0.60
Bunner (2015) [3]	81.5 (9.7)	75.8 (9.0)	−5.7 (9.4)*	87.2 (14.5)	84.4 (10.4)	−2.8 (8.8)	−2.9 (−9.2 to 3.5)	0.36
Lee (2016) [4]	75.6 (10.9)	76.7 (9.3)	1.1 (9.0)	78.1 (12.1)	76.7 (10.3)	−1.4 (9.9)	2.5	0.335
Barnard (2018) [5]	79.7 (3.8)	78.6 (3.5)	−1.2 (3.4)	76.6 (2.0)	71.9 (2.2)	−4.7 (2.5)	3.5 (−5.1 to 12.1)	0.40

Data are presented as mean (SD, if available) unless otherwise indicated, *p < 0.05, **p < 0.01, CI = Confidence Interval.

Supplementary Table S6. Changes in Total Cholesterol levels (mg/dL) by group assignment.

Reference	Intervention Group	Control Group	Between-Group Difference (Intervention-Control)	p-Value

							Mean (95% CI)	
	Baseline	Final	Change	Baseline	Final	Change		
Nicholson (1999) [1]	203.0 (42.08)	178.76 (50.97)	−24.32	214.67 (23.55)	190.35 (17.76)	−24.32	0.0	
Wheeler (2002) [6]	183.4 (7.34)	167.57 (6.95)	−15.83**	183.4 (8.88)	167.57 (8.49)	−15.83**	0.0	0.88
Barnard (2009) [2]	187.0 (5.3)	165.5 (4.5)	−21.6 (4.2)**	198.9 (6.2)	184.1 (5.5)	−14.8 (5.1)**	−6.8 (−19.9 to 6.4)	0.31
Ferdowsian (2010) [8]	186.8 (4.6)	177.1 (4.7)	−9.8 (3.6)**	183.9 (5.0)	182.3 (5.4)	−1.6 (3.5)	−8.1 (−18.6 to 2.3)	0.13
Mishra (2013) [7]	189 (4.3)	176 (4.2)	−13.7 (3.5)**	191 (3.5)	189 (3.5)	−1.3 (2.3)	−12.3 (−4.3 to −20.3)**	0.001
Bunner (2015) [3]	171.2 (48.4)	159.1 (42.6)	−12.1 (37.8)	173.4 (41.4)	175.6 (34.0)	2.2 (25.6)	−14.4 (−36.9 to 8.2)	0.20
Barnard (2018) [5]	157.4 (7.6)	146.4 (7.8)	−10.9 (6.5)	155.6 (6.0)	136.7 (7.1)	−18.9 (3.8)**	8.0 (−7.0 to 23.0)	0.28

Data are presented as mean (SD, if available) unless otherwise indicated, *p < 0.05, **p < 0.01, CI = Confidence Interval.

Supplementary Table S7. Changes in High Density Lipoprotein Cholesterol levels (mg/dL) by group assignment.

Reference	Intervention Group			Control Group			Between-Group Difference (Intervention-Control) Mean (95% CI)	*p*-Value
	Baseline	Final	Change	Baseline	Final	Change		
Nicholson (1999) [1]	44.4 (12.36)	36.68 (10.81)	−7.72*	43.24 (6.56)	42.47 (6.56)	−0.77	−6.95	
Wheeler [6]	34.36 (2.7)	35.91 (2.7)	1.55	37.07 (3.47)	34.75 (2.7)	−2.32	3.87	0.09
Barnard (2009) [2]	52.3 (2.8)	51.3 (2.6)	−1.0 (1.0)	49.8 (2.1)	48.6 (2.1)	−1.3 (1.7)	0.3 (−3.7 to 4.2)	0.89
Ferdowsian (2010) [17]	52.7 (1.6)	48.3 (1.6)	−4.3 (0.8)**	50.9 (2.1)	50.5 (2.3)	−0.4 (0.9)	−3.9 (−6.4 to −1.5)**	0.002
Mishra (2013) [7]	55 (1.8)	51 (1.7)	−3.3 (1.1)**	56 (1.4)	56.7 (1.3)	0.7 (0.9)	3.9 (−6.6 to −1.3)**	0.001
Bunner (2015) [3]	52.1 (15.1)	46.8 (11.4)	−5.2 (10.1)*	47.4 (18.9)	45.0 (16.9)	−2.4 (6.9)	−2.9 (−8.9 to 3.2)	0.34
Lee (2016) [4]	50.0 (12.3)	52.2 (14.9)	2.2 (8.8)	51.2 (13.3)	51.7 (13.0)	0.5 (8.2)	1.7	0.459
Barnard (2018) [5]	54.6 (4.6)	51.2 (4.8)	−3.4 (1.6)*	57.7 (3.8)	56.6 (3.9)	−1.1 (1.5)	−2.4 (−6.8 to 2.1)	0.29

Data are presented as mean (SD, if available) unless otherwise indicated, *p < 0.05, **p < 0.01, CI = Confidence Interval.

Supplementary Table S8. Changes in Low Density Lipoprotein Cholesterol levels (mg/dL) by group assignment.

Reference	Intervention Group			Control Group			Between-Group Difference (Intervention-Control) Mean (95% CI)	*p*-Value
	Baseline	Final	Change	Baseline	Final	Change		
Barnard (2009) [2]	104.4 (4.7)	90.9 (4.4)	−13.5 (4.3)**	117.7 (5.9)	108.3 (5.4)	−9.4 (4.5)*	−4.1 (−16.4 to 8.2)	0.51
Ferdowsian (2010) [8]	103.5 (3.9)	99.1 (4.0)	−4.4 (3.3)	106.7 (4.1)	105.3 (4.6)	−1.4 (3.2)	−3.0 (−12.5 to 6.4)	0.53
Mishra (2013) [7]	109 (3.7)	96 (3.4)	−13.0 (2.8)**	110 (3.2)	108 (3.0)	−1.7 (1.9)	−11.3 (−17.8 to −4.8)**	<0.001
Bunner (2015) [3]	89.0 (38.3)	81.2 (34.2)	−7.8 (28.9)	95.4 (43.6)	95.8 (34.5)	0.4 (21.0)	−8.2 (−25.8 to 9.5)	0.35
Lee (2016) [4]	92.7 (28.5)	89.8 (32.3)	−2.8 (17.8)	102.8 (39.0)	101.9 (38.5)	−1.0 (29.3)	−1.8	0.732
Barnard (2018) [5]	75.6 (8.1)	63.7 (7.7)	−11.9 (3.9)**	74.8 (4.9)	62.1 (4.6)	−12.7 (3.6)**	0.8 (−10.1 to 11.6)	0.89

Data are presented as mean (SD, if available) unless otherwise indicated, *p < 0.05, **p < 0.01, CI = Confidence Interval.

Supplementary Table S9. Changes in Triglycerides levels (mg/dL) by group assignment.

Reference	Intervention Group			Control Group			Between-Group Difference (Intervention-Control) Mean (95% CI)	*p*-Value
	Baseline	Final	Change	Baseline	Final	Change		
Nicholson (1999) [1]	187.61 (69.03)	165.49 (55.75)	−22.12	202.65 (169.91)	163.72 (100.88)	−38.93	16.81	
Barnard (2009) [2]	148.1 (16.1)	114.2 (9.7)	−33.9 (12.7)*	158.1 (18.8)	150.3 (28.8)	−7.8 (28.9)	−26.1 (−89.2 to 37.0)	0.41
Ferdowsian (2010) [8]	154.2 (11.8)	149.8 (10.9)	−4.4 (6.5)	131.6 (9.2)	135.2 (13.1)	3.5 (10.1)	−7.9 (−30.5 to 14.7)	0.49
Mishra (2013) [7]	129 (7.0)	143 (7.1)	13.9 (5.6)*	127 (6.28)	124 (5.9)	−2.9 (4.3)	16.8 (3.1 to 30.6)*	0.03
Bunner (2015) [3]	151(66.3)	155.7 (61.6)	4.7 (56.2)	152.6 (88.8)	174.5 (110.6)	21.9 (68.9)	−17.2 (−61.2 to 26.8)	0.43
Lee (2016) [4]	130.3 (61.7)	143.7 (92.4)	13.4 (72.8)	147.7 (113.8)	128.8 (57.9)	−18.9 (81.9)	32.3	0.053
Barnard (2018) [5]	155.9 (25.9)	176.7 (31.1)	20.8 (21.4)	115.6 (10.8)	90.7 (8.7)	−24.9 (7.1)**	45.7 (−2.1 to 93.6)	0.06
Wheeler (2002) [6] 12.5h AUC (mg%.h)	3 147 (494)	2 839 (463)	−308	3 091 (531)	2 727 (440)	−364	56	0.99

Data are presented as mean (SD, if available) unless otherwise indicated, *$p < 0.05$, **$p < 0.01$, CI = Confidence Interval.

Supplementary Table S10. Changes in Urinary Albumin/24 h (mg/24 h) by group assignment.

Reference	Intervention Group			Control Group			Between-Group Difference (Intervention-Control) Mean (95 % CI)	*p*-Value
	Baseline	Final	Change	Baseline	Final	Change		
Nicholson (1999) [1]	434.8 (565.5)	155.2 (182.6)	−279.6	82.9 (114.6)	169.2 (298)	86.3	−365.9	
Barnard (2009) [2]	33.0 (7.4)	20.2 (4.6)	−12.8 (7.0)	55.0 (37.2)	69.5 (47.2)	14.6 (12.0)	−27.4 (−55.1 to 0.3)	0.05
Barnard (2018) [5] median (mg/dL)	0.7	0.8	0.2**	0.6	0.6	−0.05	0.75 (0.1 to 9.8)*	0.027
Wheeler (2002) [6] RPF (ml.min^{-1}.1,73 m^{-2})	276 (25)	298 (30)	22	361 (43)	312 (28)	−49	71	0.21
Wheeler (2002) [6] GFR (ml.min^{-1}.1,73 m^{-2})	100 (7)	108 (8)	8	116 (9)	118 (6)	2	6	0.63
Wheeler (2002) [6] AER (µg/min)	68 (39)	49 (26)	−19	53 (29)	62 (28)	9	28	0.11

Data are presented as mean (SD, if available) unless otherwise indicated, *$p < 0.05$, **$p < 0.01$, CI = Confidence Interval.

References

1. Nicholson, A.S.; Sklar, M.; Barnard, N.D.; Gore, S.; Sullivan, R.; Browning, S. Toward Improved Management of NIDDM: A Randomized, Controlled, Pilot Intervention Using a Lowfat, Vegetarian Diet. *Prev. Med.* **1999**, *29*, 87–91, doi:10.1006/pmed.1999.0529.
2. Barnard, N.D.; Cohen, J.; A Jenkins, D.J.; Turner-McGrievy, G.; Gloede, L.; Green, A.; Ferdowsian, H. A low-fat vegan diet and a conventional diabetes diet in the treatment of type 2 diabetes: a randomized, controlled, 74-wk clinical trial. *Am. J. Clin. Nutr.* **2009**, *89*, 1588S–1596S, doi:10.3945/ajcn.2009.26736h.
3. Bunner, A.; Wells, C.L.; Gonzales, J.C.; Agarwal, U.; Bayat, E.; Barnard, N.D. A dietary intervention for chronic diabetic neuropathy pain: a randomized controlled pilot study. *Nutr. Diabetes* **2015**, *5*, e158, doi:10.1038/nutd.2015.8.
4. Lee, Y.-M.; Kim, S.-A.; Lee, I.-K.; Kim, J.-G.; Park, K.-G.; Jeong, J.-Y.; Jeon, J.-H.; Shin, J.-Y.; Lee, D.-H. Effect of a Brown Rice Based Vegan Diet and Conventional Diabetic Diet on Glycemic Control of Patients with Type 2 Diabetes: A 12-Week Randomized Clinical Trial. *PLOS ONE* **2016**, *11*, e0155918, doi:10.1371/journal.pone.0155918.
5. Barnard, N.D.; Levin, S.M.; Gloede, L.; Flores, R. Turning the Waiting Room into a Classroom: Weekly Classes Using a Vegan or a Portion-Controlled Eating Plan Improve Diabetes Control in a Randomized Translational Study. *J. Acad. Nutr. Diet.* **2018**, *118*, 1072–1079, doi:10.1016/j.jand.2017.11.017.
6. Wheeler, M.L.; Fineberg, S.E.; Fineberg, N.S.; Gibson, R.G.; Hackward, L.L. Animal versus plant protein meals in individuals with type 2 diabetes and microalbuminuria: effects on renal, glycemic, and lipid parameters. *Diabetes Care* **2002**, *25*, 1277–1282, doi:10.2337/diacare.25.8.1277.
7. Mishra, S.; Xu, J.; Agarwal, U.; Gonzales, J.R.; A Levin, S.; Barnard, N.D. A multicenter randomized controlled trial of a plant-based nutrition program to reduce body weight and cardiovascular risk in the corporate setting: the GEICO study. *Eur. J. Clin. Nutr.* **2013**, *67*, 718–724, doi:10.1038/ejcn.2013.92.
8. Ferdowsian, H.R.; Barnard, N.D.; Hoover, V.J.; Katcher, H.I.; Levin, S.M.; Green, A.A.; Cohen, J.L. A Multicomponent Intervention Reduces Body Weight and Cardiovascular Risk at a GEICO Corporate Site. *Am. J. Heal. Promot.* **2010**, *24*, 384–387, doi:10.4278/ajhp.081027-quan-255.
